# Ontological Clarity via Canonical Presentation: Electromagnetism and the Aharonov–Bohm Effect

**DOI:** 10.3390/e20060465

**Published:** 2018-06-14

**Authors:** Tim Maudlin

**Affiliations:** Department of Philosophy, New York University, New York, NY 10003, USA; twm3@nyu.edu; Tel.: +1-212-387-8172

**Keywords:** Aharonov–Bohm effect, physical ontology, nomology, interpretation, gauge freedom, Canonical Presentation

## Abstract

Quantum physics demands some radical revision of our fundamental beliefs about physical reality. We know that because there are certain verified physical phenomena—two-slit interference, the disappearance of interference upon monitoring, violations of Bell’s inequality—that have no classical analogs. But the exact nature of that revision has been under dispute since the foundation of quantum theory. I offer a method of clarifying what the commitments of a clearly formulated physical theory are, and apply it to a discussion of some options available to account for another non-classical phenomenon: the Aharonov–Bohm effect.

## 1. Introduction

Metaphysicians rightly look to physics for insight into the nature of the physical world. And once upon a time, they would get clear and articulate answers. Newton, in his Scholium on space and time, for example, beautifully conveys both the exact content of his account of Absolute Space and Absolute Time and provides the bucket argument as an empirical demonstration that the relationist theory of motion cannot be correct ([1], pp. 6–12). One can investigate whether Newton’s empirical considerations really confirm his particular account of space and time over all others (they don’t), but you know with perfect clarity what Newton thought and why.

Unfortunately, the present day situation with respect to physics and metaphysics (ontology) is nowhere in the vicinity of that clarity. Newton did not answer every ontological question one might have been interested in—first and foremost, the source of the universal force of gravitation among particles, concerning which he did not *fingere* a *hypothesis*—but Newton was both clear about some things and clear about where there was more to be said but he didn’t know what to say. Nowadays nothing is clear and sharp in the area at all. This is not news. But like the weather, everybody talks about it and nobody does anything. This paper outlines a program for the steps that might be taken.

The main aim is illustrative. It has been widely accepted that the discovery of the Aharonov–Bohm effect, in 1959, forced—or at least suggested—a radical change in our understanding of physical ontology. Briefly, the effect (once it was verified) suggested that the electromagnetic scalar and vector potentials, which were regarded as mere mathematical artifacts in the classical theory, should be regarded instead as “physically real”, while the electric and magnetic fields, which were the basic ontology of Maxwell’s theory, should be somehow ontologically downgraded. This common suggestion has been disputed, e.g., by regarding the fundamental physical ontology to be a connection on a fiber bundle, the vector potential as a means to represent the connection, and the electromagnetic field as the curvature of the bundle [2] (p. 226). However, none of the objections to it support or even permit the reversion back to Maxwell’s ontology or the Relativistic version of it: the electromagnetic field tensor.

Drawing metaphysical conclusions (or teasing metaphysical possibilities) from physics requires a certain level of clarity about just what the physical theories at issue posit. And since the advent of quantum mechanics, the practice of physics has been to relentlessly and aggressively refuse to be clear about precisely the issue that concerns the metaphysician: what exactly physically exists? In Newton’s age, this question would have been regarded as one of, if not the, central concerns of physics itself. Oddly, practicing physicists nowadays are likely to direct a student asking such questions to the philosophy department. The way this plays out in quantum theory over questions such as the status and “reality” of the wave function is well known, and I do not propose to plow that over-farmed soil yet again here. Rather, I want to pursue a similar question, as far as I can, without taking issues about the ontology of quantum theory into account. That makes the particular suggestions about ontology reached here questionable and provisional, but it should have the compensating virtue of making the methodological proposal clear and easy to follow.

What is accomplished by having a general framework in which the precise ontological commitments of a theory are made manifest and unambiguous in a disciplined way? Benefits accrue on both the conceptual and heuristic sides. Conceptually, one requires this sort of clarity to truly understand a physical theory. Without it, one can do calculations and produce predictions, but not be clear about what kind of physical world the theory presents. Quantum theory, as a whole, presents an excellent example. As for developing new theories, we will see that the method of presentation suggests almost algorithmic ways to alter the ontology of a theory.

With that as prelude, then, let’s begin.

## 2. What is a Physical Theory?

Let’s start by zooming out all the way to the most general question we can ask: what is physics? Back in the day, physics was characterized as the theory of matter in motion. That remains a wonderful place to start, although the slogan needs some tweaks and updates. One nice thing about the slogan is that it immediately indicates the “foundational” aspect of physics as opposed to all the other empirical sciences. Every empirical science—biology, geology, psychology, economics, etc.—deals with systems that are matter in motion. A living horse, whatever else it is, is matter in motion and, as such, falls under the purview of physics. It also falls under the purview of biology, evolutionary theory, economics, cognitive science, and so on. But the converse does not hold: not every physical system provides subject matter for biology or geology or psychology or economics. A red dwarf star, for example, does not. However, the red dwarf is matter in motion and must be susceptible to physical analysis.

The phrase “matter in motion” offers two targets for conceptual and physical analysis: matter and motion. In contemporary physics, there is no such objective state as “being in motion”. A particle in interstellar space, for example, can be “at rest” in the sense of not moving relative to its own inertial frame and at the same time “moving” relative to other perfectly legitimate inertial frames. Since the theory of Relativity, talk of the “motion of a system” has come to be understood as talk of the trajectory of the system through space-time. If you specify a space-time structure and the worldlines of the constituents of the system, then you have specified all there is to say about the “motion” of the system.

Of course, in order to *have* a trajectory through space-time, a worldline, the constituent has to be a *local beable* in John Bell’s sense [3]. That is, the constituent has to be something that has a reasonably well-defined location in space-time. And there may exist items in the physical ontology of a theory that fail to have this characteristic. Those would be the *non-local beables* of the theory. In quantum theory, the *quantum state* (the item represented by the wave function) is such a non-local beable according to most explications of the theory. Hence there arises a trivial semantic issue: should the non-local beables of a theory count as *matter* or as something else, some *tertium quid* beside the space-time and the local beables?

Nothing hangs in this semantic decision, but for the sake of clarity I will, in this paper, refer only to local beables as “matter”. According to this usage, the quantum state, if it exists at all, is an immaterial and non-local physical item. If the reader dislikes this terminology, she is invited to systematically rewrite it in whatever way is congenial.

Adopting this updated terminology, then, the fundamental ontology of any physical theory contains a space-time structure, some local beables (matter), and whatever else may have come up. Physics, the theory of matter in motion, has become the theory of the trajectories of local beables through space-time.

In the course of theorizing, we may increase the ontology of a theory by postulating some new items, local or nonlocal. Methodologically, the grounds for doing that is typically in service of accounting for the motions of a particular set of local beables, the postulated constituents of familiar observable matter. Thus, for example, Maxwell introduced the electric and magnetic fields as novel local beables in order to account for the motions of observable objects such as iron filings. By our convention, these fields are material because they are local beables. On the other hand, the postulation of a quantum state, which is a non-local beable, counts as adding an immaterial entity. It is, in any case, certainly an entity that cannot be directly observed. The only grounds we have to believe in quantum states is the influence they have on observable collections of local beables such as those that constitute pointers and radios and tables and chairs.

To sum up so far, and present this account in more detail: physics is the description of *local beables and whatever else is required to account for their trajectories through space-time*. That updates the more mellifluous “Matter in motion” in a way useful for present purposes. Another, essentially equivalent characterization of physics is *the most general account of what there is and what it does, at the fundamental level*. The “what there is” part is provided by the *ontology* of the theory, and the “what it does” part by the *dynamics*. The restriction to fundamental items excludes treating derivative entities, such as horses or species or economic systems, as the basic subject matter.

Specifying both what there is and what it does has, throughout history, involved specification of a space-time structure in which the behavior occurs. Whether the spatiotemporal structure is substantival, or relational, or something else, we do not prejudge. For the purposes of this paper, we will consider mostly classical space-times, but that is just an historical accident.

We now have four basic “categories of being” postulated by a physical theory:

(1) The local beables or matter, which exists at delimited regions of the spatio-temporal structure.
(2) The non-local beables (if any) that have no particular value at any space-time location.
(3) The spatio-temporal structure, in terms of which the distinction between local and non-local beables is drawn.
(4) The dynamical laws, which specify, either deterministically or probabilistically, how the various beables must or can behave.

There is no requirement that the elements of each of these categories be definable independently of the rest. Indeed, differentiating the local from the non-local beables obviously requires reference to the spatio-temporal structure. Nor is there any transcendental argument that these four categories of being exhaust the whole of physical reality, or that all of them must be exemplified. Einstein, for example, famously opined that the progress of physics marched inexorably toward locality, in the ontology and even in the laws:

If one asks what, irrespective of quantum mechanics, is characteristic of the world of ideas of physics, one is first of all struck by the following: the concepts of physics relate to a real outside world, that is, ideas are established relating to things such as bodies, fields, etc., which claim “real existence” that is independent of the perceiving subject—ideas which, on the other hand, have been brought into as secure a relationship as possible with the sense data. It is further characteristic of these physical objects that they are thought of as arranged in a space-time continuum. An essential aspect of this arrangement of things in physics is that they lay claim, at a certain time, to an existence independent of one another, provided these objects “are situated in different parts of space”… This principle has been carried to extremes in the field theory by localizing the elementary objects on which it is based and which exist independently of each other, as well as the elementary laws which have been postulated for it, in the infinitely small (four-dimensional) elements of space.

[4] (pp. 170–171)

All of our four categories of being have been subject to intense philosophical scrutiny through the ages. The nature of space and time has already been mentioned. The nature of matter has been equally treated in fundamentally different way: as that which occupies space; as the center of forces; as a bundle of properties, etc. Laws, of course, have been rendered as relations of necessitation, as members of the simplest and strongest axiomatization of the Humean mosaic, as dispositions and as primitive entities not subject to further analysis. For our purposes here, though, these controversies are inessential: one way or another every physical theory does postulate a dynamics or, as we will say, a *nomology*. Just as the ontology of a theory specifies what exists in a more “concrete” sense, the nomology specifies the laws. The spatiotemporal structure has traditionally been taken to be somehow less than material but more than nothing. And the non-local ontology is a recent innovation in physics, still a matter of much dispute (See, e.g., [5]).

Let’s try a couple of simple exercises, drawn from antiquity. Democritean atomism has a material ontology of solid, shaped, indivisible, and solid bodies. The spatiotemporal structure is an infinite Euclidean space that persists through time, with the opposite directions “up” and “down” intrinsic to it. The nomology specifies that the atoms will fall at a constant rate down, save for two circumstances: collisions between atoms and the occasional random “swerve”. The timing and nature of these swerves are not precisely defined by Democritus, nor are the laws of collision.

Aristotelian physics, in contrast, posits a finite, spherical spatial structure that persists through time. There are five fundamental types of matter: earth, water, air, and fire (which correspond to the modern notion of the states of matter: solid, liquid, gas and plasma), as well as the quintessence, viz. aether. The spherical spatial structure defines the “up” and “down” directions as towards and away from the center of the universe. The dynamics is given in terms of nature rather than in terms of laws: the natural motions of earth and water are to the center and the natural motions of fire and air to the periphery. The natural motion of aether is uniform circular motion about an axis through the center of the universe.

The four basic categories used in our anatomy of a physical theory are to be taken *cum grano salis*. The category of non-local beables is a recent addition, and there may be more to come. In the other direction, one could imagine categories melding: the spatio-temporal and material aspects, for example, becoming so entwined as not to always be distinct. But at least in some conditions the separate categories must emerge if the theory is to be recognizable as physics at all.

The most profound conceptual addition to this basic scheme of physical theorizing is strongly associated with the scientific revolution: the geometrization or mathematization of physics. Galileo famously declared:

Philosophy is written in this grand book—I mean the universe—which stands continually open to our gaze, but it cannot be understood unless one first learns to comprehend the language in which it is written. It is written in the language of mathematics, and its characters are triangles, circles, and other geometric figures, without which it is humanly impossible to understand a single word of it; without these, one is wandering about in a dark labyrinth.

[6] (p. 65)

Modern physics cannot be separated from this mathematization. Our next task is to consider the use of mathematics in physics and how to cope with it.

## 3. Mathematical Physics and the Canonical Presentation

As the simple examples of Democritus and Aristotle illustrate, a physical theory need not employ any sophisticated mathematical apparatus. However, both the glory and the bane of modern physics is its highly mathematical character. This has provided both for the calculation of stunningly precise predictions and for the endemic unclarity about the physical ontology being postulated. The unclarity arises from a systematic ambiguity among terms that refer to the ontology and nomology of a theory and terms that refer to the *mathematical representations* of the ontology and nomology.

Mathematical physics uses mathematical structures to represent physical states of affairs. Unfortunately, the distinction between the representations and the entities represented is often elided in the common manner of speech. As a simple example, take the term “scalar field”. It would not sound in the least odd to say: “The Higgs field is a scalar field”. Further, if one inquires what a scalar field is, it would not be at all out of place to be told that a scalar field is a mapping from space-time points into a set of scalars, i.e., real or complex numbers. However, if we naively put these two innocuous statements together we appear to get the result that the Higgs field (which we took to be a physical item) is a mapping from space-time points into the field of numbers (which is an abstract mathematical object). As a purely abstract matter, infinitely many such mappings exist, but that does not mean there are infinitely many physical scalar fields. Something has gone wrong.

On the other hand, to take a familiar example, we often talk of the wave function of a system in quantum mechanics, and ask whether the wave function is “real”, “physical”, or “objective”. However, what is a wave function? As the term implies, it is a *function*, a mathematical mapping from (e.g.) the configuration space of a system to the complex numbers. So is the dispute about the “reality” of the wave function a dispute about the ontological status of such a mapping? Obviously not.

What has gone wrong in both of these examples is straightforward: the terms “scalar field” and “wave function” are being used ambiguously. In one sense, they refer to a specific *mathematical structure*. In the other, they are used to refer to *a postulated physical item that is supposed to be represented by the mathematical structure*. It is trivial that the mathematical structure exists in whatever sense mathematical entities do. It can be highly contentious whether any physical entity exists that can be represented by that mathematical item in the way that the physical theory requires.

The most efficient way to resolve any systematic ambiguity is by a linguistic convention. In the case of the “wave function”, I have adopted the convention that the mathematical representation shall be called a “wave function” (because a *function* is plainly a mathematical item) and the non-local beable that it is posited to represent shall be called the *quantum state* of a system. So the metaphysical battles are over the existence and nature (if they exist) of quantum states.

There is no canonical way for a mathematical item to represent a physical one. No amount of staring at the mathematics per se can resolve questions like: which mathematical degrees of freedom in the representation correspond to physical degrees of freedom in the system represented? Mathematical degrees of freedom in the representation that do not correspond to physical degrees of freedom in the represented system are called *gauge degrees of freedom*, and representations that differ only in their gauge degrees of freedom are called *gauge equivalent*. Transformations between gauge equivalent mathematical representations are called *gauge transformations*.

As a seemingly trivial example, a global constant change in the phase of a wave function is standardly taken to be a gauge transformation and the resulting mathematical representation to be gauge equivalent to the original. One way to remove this redundancy from the mathematical representations is to change to another mathematical object as the vehicle of representation. In this case, one says that quantum states are not properly represented by *vectors* in a Hilbert space but by *rays* in a projective Hilbert space. In one sense of “ideal”, an ideal mathematical representation for a physical system would implement a one-to-one map from the space of mathematical representations to the space of physically possible states of the system. At least, such a representation would be maximally convenient for the metaphysician, while it might be severely impractical for the physicist who actually has to compute numbers. We will see an example of this anon.

If one cannot distinguish the gauge from the non-gauge degrees of freedom in a mathematical representation by any purely mathematical analysis, how is that job to be done? The only way is by a *commentary* on the mathematical representation. We can only be sure what a piece of mathematics is supposed to represent (if anything) by being told by the expositor of a physical theory. *One and the same mathematical apparatus accompanied by a different commentary can convey different physical theories, theories with different ontologies and even with different laws*. Our examples from classical electromagnetism will illustrate this possibility.

Although this paper is not particularly about quantum theory, it is worthwhile to pause for a moment to reflect on the situation there. It is commonly said that there is this thing called “quantum theory” which works splendidly well as a physical theory but nonetheless lacks an “interpretation”. The project of interpreting quantum theory is assigned to (or dumped on) people who work on the foundations of physics, and perhaps most usually to philosophers. “Interpreting” quantum theory is regarded by many physicists as pointless or frivolous or unscientific or even meaningless, except insofar as it means altering the empirical predictions of the theory. This attitude has a long history: already in 1926 the physicist Charles Galton Darwin wrote to Niels Bohr: “It is a part of my doctrine that the details of a physicist’s philosophy do not matter much” [7] (p. 4).

According to the linguistic usage urged here, though, all of this talk of “interpreting” quantum theory is mistaken. If a physical theory is supposed to address the questions of what there is and what it does, the questions of physical ontology and physical nomology, then what goes by the name “quantum theory” is not a theory at all. It is rather a mathematical method of making predictions. If all one cares about are the accuracy of the predictions, then one can be completely satisfied with “quantum theory”, but that is the attitude of the engineer rather than the natural philosopher. “Quantum theory” is a prediction-making recipe in need of a real physical theory, a theory that specifies what exists and how it behaves, thereby accounting for the remarkable reliability of the predictive algorithm. Rather than there being a theory in need of an interpretation, there is a calculational tool in need of a theory that accounts for it.

How, then, is one to clearly and unambiguously specify a physical theory? And what difference might it make to have our physical theories clearly and unambiguously presented? The most important elements of a mathematically formulated physical theory have already been given, and all that is required is a systematic way to exhibit them. What we need to make clear are the *physical ontology*, the *spatiotemporal structure,* and the *nomology* (i.e., the fundamental laws) of the theory, by means of presenting the *mathematical representation* of these various elements along with a *commentary* that relates the mathematical representation to the physical item it is meant to represent. The commentary should make clear which mathematical degrees of freedom in the representation are *gauge* and which rather correspond to *physical degrees of freedom* in the system represented. In addition, it can be useful to specify any *mathematical fictions* that may be employed for the purpose of simplicity of presentation or of calculation, as well as any *derivative ontology*, i.e., physically real items that are composed of and analyzable into more fundamental entities. To take an obvious example, hydrogen atoms are real physical entities, but they are not physically fundamental: they are just bound states of a proton and an electron. If protons and electrons are already in one’s physical ontology and the nomology allows for them to form bound states, then the recognition of hydrogen atoms as physically real does not increase one’s ontology at all.

In presenting a clear and precisely articulated physical theory, then, one ought to specify the fundamental physical ontology and how the fundamental physical ontology is to be represented mathematically. The fundamental nomology—the laws—will be represented by mathematical equations that make reference to the spatiotemporal structure, and we demand that these mathematical representations of the laws contain *only representations of the fundamental ontology*. Hydrogen atoms may be physically real, but the laws of nature do not influence them qua hydrogen atoms but qua electron and proton (or better: electron and quarks) that happen to form a bound state. Carrying this insight further, we see that there can be no fundamental physical laws that mention tables or chairs or horses or economic systems or measurements *as such*. Physics deals with these items only as derivative ontology, not as fundamental ontology. The special sciences can usefully then be regarded as operating under the fiction that the sorts of items they trade in are fundamental rather than derivative, and are governed by sui generis laws. Ultimately, physics must explain the predictive effectiveness of these special science laws, just as it must explain the predictive effectiveness of the quantum predictive algorithm.

Collecting together all of these threads, we can at last make a concrete proposal. A *Canonical Presentation* of a mathematical physical theory shall specify:

(1) The fundamental physical ontology of the theory, which may further be divided into the local beables (matter and space-time structure) and the non-local beables, if any (e.g., a quantum state represented by a wave function on configuration space).
(2) The spatio-temporal structure of the theory.
(3) The mathematical items that will be used to represent both 1 and 2, with a commentary making clear which degrees of freedom in the mathematics are gauge and which are not.
(4) The nomology of the theory, which will be represented by equations couched in terms only of the items mentioned in (3).
(5) Mathematical fictions—these are mathematically defined quantities that are not intended to directly represent any part of the physical ontology. Such fictions can play an important practical role when trying to calculate with the theory.
(6) Derivative ontology—these are items that are taken to be physically real but not fundamental. They must be definable in terms of the fundamental ontology and nomology.

The only way to argue for the utility of this sort of systematic presentation of a physical theory is to see it in action. That is our next task.

## 4. The Canonical Presentation of Classical Electromagnetic Theory

Let’s see how this general approach to specifying a theory in mathematical physics works in a relatively uncontroversial setting: classical electromagnetism. Even there, we will confront some interesting and perhaps unexpected choices when trying to lay out the theory in such an explicit way.

Expressed in words, classical electromagnetic theory, as codified in Maxwell’s equations, posits the existence of an electric field, a magnetic field, matter with mass and charge, a classical space-time, and several laws.

A naïve Canonical Presentation of the physical theory, drawn straight from a standard textbook, might look like Table 1.

In presenting the nomology, we have adopted Metaphysician’s Units: the speed of light c and 4π have both been set to unity. We have allowed ourselves the liberty because for our purposes now those quantities just clutter the Presentation up.

The Canonical Presentation makes some things immediately clear. We are considering a theory in which the electric and magnetic fields are two distinct physical items, each represented by a vector field on a Euclidean space. The Lorentz force, and hence forces in general, are taken to be real parts of the physical ontology. They, like all the rest of the physical ontology, are local beables. We could provide more detail about the mathematical structure of the mathematical representations, some of which is easy to fill in. For example, both ρ and μ are functions defined over R^3^ for each value of t. t ranges over some interval of the real numbers.

Looking carefully at the nomology, we see that we have not quite managed to satisfy all of the requirements of a Canonical Presentation. In particular, the mathematical term $\vec{v}$ appears thrice in the nomology but is not the mathematical representation of any part of the ontology. Intuitively, $\vec{v}$ is the velocity of the charged matter density, and one would think that specifying μ as a function of t would serve to fix $\vec{v}$. However, because μ is supposed to be a continuous matter *density*, this is just not so. For example, suppose μ is the same for all t: a positive constant inside a sphere in E^3^ and zero outside it. One might immediately assume that this is the mathematical representation of a uniform sphere of matter at rest. However, a moment’s thought reveals that it could just as well represent a uniform sphere of matter rotating on an axis, or performing any other motion that an incompressible continuous fluid might. In short, the physical content expressed by the function $\vec{v}$(x, y, z, t) outruns the physical content expressed by μ(x, y, z, t). If one really wants to make a matter density on a continuum a fundamental part of the physical ontology, then one must also accept that there is a velocity function assigned to the matter density that does not supervene on the distribution of the matter density over all of space for all of time.

The theory also has as yet no mechanism to implement conservation of charge or conservation of matter. Conservation of charge is easy: add a law to the effect that $\frac{\partial \vec{J}}{\partial t}$ + Div($\vec{J}$) = 0. Conservation of matter would require a similar law: $\frac{\partial \mu \vec{v}}{\partial t}$ + Div($\mu \vec{v}$) = 0.

It might also strike one as odd that the physical ontology contains both a matter density and a charge density with nothing to link them together, either in virtue of a definition or in virtue of a law. As far as our principles tell us, there could be a positive charge density where the matter density is zero, just as there could be positive matter density where the charge density is zero. The latter represents something we understand: the possibility of uncharged matter. The former, though, makes no obvious sense as it represents, as it were, free-floating charge.

Both of these problems are soluble by changing the theory from one with a matter density ontology to one with a point particle ontology. A point particle has a precise location at every moment of time that it exists. If we require that point particles neither be created nor destroyed and that they always move continuously, then the spatio-temporal career of every point particle will be represented by a world line: a continuous path through space as a function of time (or, relative to a different set of representational conventions, a continuous path through space-time). Charge conservation is secured by simply associating a quantity, the charge, with each particle, and similarly for mass. The Canonical Presentation of this new theory would be as seen in Table 2.

$\vec{v_{i}}$ is now rigorously defined, and we understand why there cannot be charge where there is no matter: charges are ascribed to particles and so can only exist where particles do, and particles must also have non-zero mass. But some of our mathematical issues have just been moved around to different places: officially, $\mathrm{Div}\left(\vec{E}\right)$ cannot be well-defined if the charge is only non-zero at a point. So to get our ducks in order we would have to massage the Presentation even more. But since our main concern here is with the status of the electric and magnetic fields, not with the mass density or the particles, we will not pursue that problem further. We only pause to note that getting one’s ducks in a row requires first figuring out which ducks have strayed, and that the discipline we have imposed on ourselves by trying to present the theory in the form of a Canonical Presentation brings those delinquent ducklings into focus.

Let us turn our attention now to the nomology of the particle theory. There are a surprising number of “laws of nature” in this presentation: six just for classical electromagnetism with charged point particles. The ideal of a completed physics is usually envisaged as a single equation that covers everything, so compact as to fit on the proverbial T-shirt. Surely we can do better than this unruly crowd of a half-dozen. In terms of our Canonical Presentation, we would like to depopulate the nomology. There are several different sorts of moves that can accomplish this.

The easiest case to consider is the “law” $\mathrm{Div}\left(\vec{B}\right)=\;0$. The content of this equation is often described as saying that there are no magnetic monopoles. But the proposition “There are no magnetic monopoles” does not really have the form of a paradigmatic law. Consider, for comparison, the proposition that water is H_2_O. That is certainly true, and necessarily true, but does not have the characteristics of a physical or chemical law. We do not think that there are as many distinct chemical laws as there are chemical species: that would lead to millions of laws of chemistry. Water is H_2_O is rather the answer to the fundamental philosophical question: What is it? It tells of the *metaphysical nature* of water, what water fundamentally is. Although different in some respects, “There are no magnetic monopoles” is a similar sort of claim: it specifies part of the fundamental nature of magnetic charges.

Still, to make the connection between the absence of magnetic monopoles and $\mathrm{Div}\left(\vec{B}\right)=\;0$, we need a connection between the divergence of the magnetic field and magnetic charges. What would cut this Gordian knot is another statement about the fundamental metaphysical nature of electric and magnetic charges: what if magnetic charges *just are* the divergences of magnetic fields, and electric charges *just are* the divergences of electric fields? Then the ontological claim that there are no magnetic monopoles is completely equivalent ontologically to the claim that the magnetic field is divergenceless, which in turn is *represented* by the mathematical equation $\mathrm{Div}\left(\vec{B}\right)=\;0$. The net result of this ontological move is to shift the equation $\mathrm{Div}\left(\vec{B}\right)=\;0$ from the category of the nomology to a logical consequence of an ontological analysis of the form $\mathrm{Div}\left(\vec{B}\right)=$ q_m_, with q_m_ representing a magnetic charge, together with the negative ontological claim that magnetic charges do not exist.

A non-existence claim such as this is the sort of beast that drove Parmenides around the bend, and there are various methods we might use to incorporate it into our Canonical Presentation. But the most elegant is a simple rule of silence: in a physical theory nothing exists unless we explicitly say it does. So the elegant way to deny the existence of magnetic charges is simply not to list magnetic charges in the ontology.

Electric charges, though, do exist. But we can eliminate $\mathrm{Div}\left(\vec{E}\right)={\;q}_{i}$ from the nomology by exactly the same shift. Let us propose that this equation represents not a physical law but an ontological analysis: electric charges *just are* the divergences of electric fields. In this way we reduce both the physical ontology and the nomology, and further gain an explanation of why electric charges cannot exist without electric fields.

Bundling these two changes together (one could in principle do just one or the other alone) yields a new, different physical theory (Table 3).

If the method of handling $\mathrm{Div}\left(\vec{B}\right)=\;0$ strikes one as too baroque, there is another tack available. Simply stipulate that magnetic fields are to be represented mathematically by divergence-free vector fields. There is nothing preventing this sort of decision, which now appears as a further restriction on the mathematical apparatus (Table 4).

Note that although this Canonical Presentation is not identical to the last, a strong argument can be made that they present one and the same physical theory. The two presentations have identical physical ontologies, identical spatiotemporal structures and identical nomologies. Their methods of handling magnetic monopoles can therefore be reasonably regarded as *merely* presentational differences, to which no ontological fact corresponds.

There are a couple more mathematical worries one might have about particle ontology electromagnetic theories. If the charge of a particle is concentrated at a point, then the electric field will not be defined there. That raises problems for both the divergence in $\mathrm{Div}\left(\vec{E}\right)=\;q_{i}$ and for the equation for the Lorentz force on the particle. There are different mathematical approaches to solving problems like this, but for illustration’s sake here is a sketch of one.

$\mathrm{Div}\left(\vec{E}\right)$ is problematic at every point along the worldline of the particle—exactly the points where the charged particle exists! But we can take a page from Gauss to circumvent this problem. Gauss’s theorem says that the integral of the flux of a vector field over a closed surface equals the integral of the divergence over the enclosed volume. And there is no problem defining the flux over any surface enclosing the particle. So instead of defining q_i_ as the divergence of the electric field at a point, we can define it as the limit of the flux over surfaces that enclose the point as the maximum distance from the point to the surface goes to zero. In a similar spirit, define the *q_i_-adjusted electric field* E_qi_ at a point p as the electric field at p minus $\frac{q_{i}}{r^{2}}\hat{r}$, where r is the distance from the location of particle i to p and $\hat{r}$ is the unit vector in the direction from the location of the particle to p. Let $\bar{E_{\mathrm{qi},\;\Sigma }}$ be the average of E_qi_ over the surface Σ. Finally, define the electric field at a point on the worldline of particle i to be the limit of $\bar{E_{\mathrm{qi},\;\Sigma }}$ as the distance of the points on Σ from the point on the worldline goes to zero. It is that value of E that is used in the Lorentz force law.

What are the solutions to these dynamical equations like? I make no representation that they give the correct results. I have rather just been illustrating the sorts of conceptual, technical, and mathematical issues that have to be resolved to complete the Canonical Presentation. It forces one to answer questions about the ontology of a theory, the nomology, and the way the mathematics is being used to represent both. Those questions, in turn are illuminating and suggestive.

The last and most important thing to note in all of these Canonical Presentations is the status of the vector and scalar potentials. When one learns classical electromagnetism, the ontological status of these potentials is not in doubt: they are not “real”, meaning that there is nothing in the physical ontology that they directly represent. They can, of course, indirectly represent the electric and magnetic fields, which are real, but the representation relation is indirect. It goes through the mathematical truths listed on the Presentation, which imply the well-known gauge freedom in choosing the potentials. It is that very freedom—the fact that there are mathematical degrees of freedom in the representation that *do not* correspond to physical degrees of freedom in the object represented—that makes the potentials so useful. One is free to choose a convenient gauge, different for different circumstances, that makes the mathematics easier to handle. One of the most important entries in our charts so far, then, is the characterization of the potentials as mathematical fictions. So far we have been considering moves—changes in the fundamental ontology of the theory—that can shift entries out of the nomology category and the ontology category and so reduce the basic posits of the physics. However, the theory of electromagnetism had to deal with a shocking empirical discovery that shifted in the other direction: from mathematical fiction to ontological posit. That is the next chapter of our tale.

## 5. The Aharonov–Bohm Effect

The physical theories presented above are not capable—even in principle—of accounting for some observable results that can be obtained in the lab. It is true that these results were not discovered at random: they were predicted by quantum theory and then confirmed. However, there is no reason in principle why they could not have been stumbled across. Since the illustrative points I want to make would become too bogged down if we tried to deal with quantum theory here, I will ignore all the theoretical details and just focus on the phenomenon and the trouble it causes.

**Figure 1:**
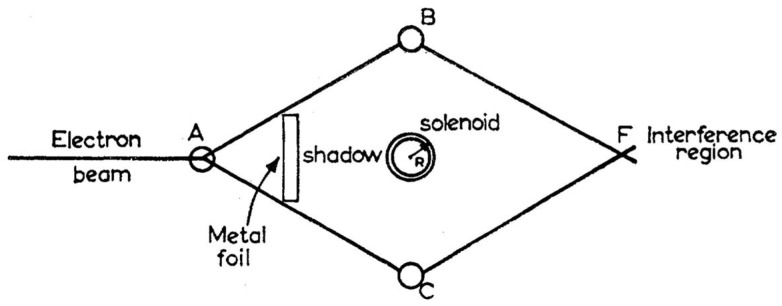
The Aharonov–Bohm set-up.

The story is well known. There is both an electric and a magnetic Aharonov–Bohm effect described in [8], but the magnetic is more familiar and certainly striking enough. Take the usual two-slit set-up for demonstrating interference effects with electrons and embed a solenoid between the two slits. Shield the solenoid so no magnetic field inside leaks out and no electrons from outside can penetrate in. Run the experiment and note where the interference bands are formed. Then change the magnetic flux in the solenoid *without altering either the electric or magnetic fields outside the solenoid.* Run the experiment again. The interference bands will be found to have shifted. The exact amount of the shift as a function of the flux in the solenoid can be calculated and the prediction checked. They match. Figure 1 shows the experimental set-up as depicted in the original paper of Aharonov and Bohm [8] (p. 486).

It is clear from our Canonical Presentations above that none of those theories are capable, even in principle, of accounting for the effect if the experimental conditions are as described. Note that everything in these theories—both the ontology and the nomology—is local in Einstein’s sense. There are no non-local beables, and the equations in the nomology take the form of local differential equations. That means the if there are no changes in the experimental conditions outside of the solenoid on the two trials of the experiment, then there can be no changes in the outcome of the experiment outside of the solenoid either. But changing the flux inside the solenoid, according to the theory, does not alter either the electric or the magnetic field outside. And the electrons themselves cannot penetrate the solenoid to be affected. In principle, an action-at-a-distance theory could account for the difference in outcomes, but none of the laws in any of the nomologies are action-at-a-distance laws. So none of our ways of articulating classical electromagnetic theory is up to the task of explaining the effect.

How was the effect originally predicted? The quantum state of the electron gets coupled to the *vector potential*, not to the electric and magnetic fields. Furthermore, when the magnetic field inside the solenoid changes, the potentials *outside* the solenoid have to change as well. This follows from the Generalized Stokes Theorem: the integral of a one-form over the surface enclosing a volume must equal the integral over the enclosed volume of the exterior derivative of the one-form. In three dimensions, as stated in the more familiar vector calculus, this amounts to the claim that the integral of a vector field over the surface of a volume equals the integral over the enclosed interior of the Curl of the vector field. Now as noted in the Canonical Presentations above, since the divergence of the magnetic field $\vec{B}$ is zero, there exists a vector field $\vec{A}$ such that $\vec{B}=\mathrm{Curl}\left(\vec{A}\right)$. So if we change $\vec{B}$ inside the solenoid, we change $\mathrm{Curl}\left(\vec{A}\right)$ inside the solenoid, and hence we change $\mathrm{Curl}\left(\vec{A}\right)$ in the interior of any closed surface that contains the solenoid. However, by the Generalized Stokes Theorem that means that $\vec{A}$ must change on the surface of the volume, even if the surface lies as far as you like from the solenoid. Changing $\vec{B}$ inside forces a change in $\vec{A}$ outside, even though $\vec{B}$ may not change a bit outside. And since the electron wavefunction couples to $\vec{A}$, this allows there to be local laws that predict the shift in the interference bands so long as those laws advert to $\vec{A}$, rather than to $\vec{B}$.

But according to our rules, $\vec{A}$ can only be used in specifying the nomology if it appears in the ontology. So the obvious way to try to account for the Aharonov–Bohm effect is to *move the scalar and vector fields from the category of Mathematical Fictions to the category of Physical Ontology.* This is the formal implementation in the setting of Canonical Presentations of the moral that Aharonov and Bohm draw in their paper. The title is straightforward: “Significance of Electromagnetic Potentials in Electromagnetic Theory”. The paper opens ([8], p. 485):

In classical electrodynamics, the vector and scalar potentials were first introduced as a convenient mathematical aid for calculating the fields. It is true that in order to obtain a classical canonical formalism, the potentials are needed. Nevertheless, the fundamental equations of motion can always be expressed directly in terms of the fields alone.

In the quantum mechanics, however, the canonical formalism is necessary, and as a result, the potentials cannot be eliminated from the basic equations. Nevertheless, these equations, as well as the physical quantities, are all gauge invariant; so that it may seem that even in quantum mechanics, the potentials themselves have no independent significance.

In this paper we shall show that the above conclusions are not correct and that a further interpretation of the potentials is needed in quantum mechanics.

Colloquially, this change in the status of the potentials is often reported by saying that in quantum theory, but not in the classical Maxwell theory, the scalar and vector potentials are physical, physically real, or real.

The Canonical Presentation offers a direct way to indicate this change: move the two potentials from the category of Mathematical Fictions to the category of Physical Ontology. There is no obvious need to make them non-local beables, so we won’t.

Having added the potentials to the Physical Ontology, there are then a series of other decisions to make, which can be made in many ways. The first one is whether the addition of the potentials to the ontology should be accompanied by the elimination of the electric and magnetic fields.

Were we to leave the fields in place there would be several consequences. One is simply that the ontology becomes more bloated, presumably to Ockham’s consternation. However, that is the least of it. The real problem is that the potentials and the fields are not independent degrees of physical freedom: they cannot vary independently of each other. In order to avoid such dependent behavior, we would have to add a new law to the nomology. In particular, the relation $\vec{B}=\mathrm{Curl}\left(\vec{A}\right)$, which has so far appeared only as a mathematical observation, would have to become a law relating two distinct physical magnitudes. While Ockham is known for trying to reduce the ontology to the smallest possible set, our main goal has been to reduce the *nomology* to just one compelling equation. Any sort of behavior can be accounted for by any ontology if you allow yourself enough laws, but that is not the case given a single fixed law, even if you increase the ontology.

There is one obvious new home for $\vec{B}=\mathrm{Curl}\left(\vec{A}\right)$: a constitutive definition of the magnetic field in terms of the vector potential. In this picture, magnetic fields are *real* but also *derivative*: magnetic fields are, as it were, *made out of* or *aspects of* vector potentials. As derivative, they come with no ontological cost. Since the equation is now just part of a (real) definition, they come with no nomological cost either. They are, in a sense, descriptive conveniences as the potentials were originally calculational conveniences. However, while the original potentials did not exist at all, in the new scheme the fields do exist as derivative physical entities. These derivative entities will certainly obey Humean laws: the very laws of Maxwell. However, just as the ontology is derivative and comes at no cost, so too does that Humean nomology.

The same move can be made by swapping out the scalar potential for the electric field. However, having made the potentials “real”, there are knock-on consequences for the nomology. Recall that the mathematical expression of the Nomology should be couched in only in terms of the Physical Ontology and the Spatiotemporal Structure. So the reference to $\vec{E}$ and $\vec{B}$ must be expunged in favor of $\vec{A}$ and ϕ. Rewriting Maxwell’s equations in terms of the potentials was the bread and butter of physicists using the classical theory because, as we have mentioned, the gauge freedom of the potentials offered opportunities to simplify calculations. If we do nothing more than rewrite the equations in this way and return from the point particle ontology to the mass density ontology, we get this Canonical Presentation (Table 5).

This theory has the same problem with the $\vec{v}$ term in the nomology as our original theory did. However, a much more severe issue has arisen: the new dynamics of $\vec{A}$ and ϕ is now radically indeterministic. The gauge freedom so prized by mathematicians has been converted into a real unconstrained physical freedom. Given any solution of the dynamical equations with a given set of initial conditions one can generate a physically different solution with the same initial conditions. Simply choose an arbitrary function ζ(x,y,z,t) whose initial gradient is zero and initial time derivative is zero. Plugging that into the equations for what used to be a gauge transformation yields a new solution from the same initial state.

The Canonical Presentation above is the most naïve and ham-handed way to implement the command to “regard the scalar and vector potentials as real”. It illustrates the dangers of mindlessly reifying some mathematical object to serve some end. It is true that according to the theory when the physical state in the solenoid changes (by changing the $\vec{A}$ field so that it’s Curl changes), the state of $\vec{A}$ out in the region available to the electron also changes. It becomes possible, then, to account for the phenomena with a theory that posits only local interactions. However, the radical indeterminism is surely too high a price to pay, especially when the phenomena themselves display no indeterminism or unpredictability (at the level of the location of the interference bands).

The problem is that we now have more physical degrees of freedom than are required for explanatory purposes. The solution is to posit some new constraint or restriction that kills off that surplus physical degree of freedom. In classical electromagnetic theory, carried out using the potentials, this would be called “fixing a gauge”, but keep in mind that in that theory nothing at all physical was at stake. The most convenient gauge for each problem could be chosen, varying the choice from one situation to the next. However, exactly because of the practical utility of finding a convenient gauge, various gauge-fixing conditions were developed. Our job now is to consider what happens if we use one or another of these conditions to eliminate the indeterminism. What physical features do these various theories have?

The first, and most famous, gauge is Lorenz gauge (after Ludvig Lorenz). Lorenz gauge kills off some of the extra degrees of freedom by requiring that $\mathrm{Div}\vec{A}=\;-\frac{\partial \phi }{\partial t}$. This condition clearly simplifies the nomology, in that the second equation becomes $\nabla^{2}\vec{A}-\frac{\partial^{2}\vec{A}}{\partial t^{2}}=\rho \vec{v}$. That has two notable effects. First, it separates the variables, so there is one equation that mentions only the scalar potential and another than mentions only the vector potential. Second, the two equations have the same form, namely $\nabla^{2}X-\frac{\partial^{2}\vec{X}}{\partial t^{2}}=Y$ or ☐^2^ X = Y.

Here is the Canonical Presentation (Table 6).

This method of gauge-fixing does not kill off the gauge degrees of freedom completely. In addition, one must put a constraint on how the scalar field behaves as it goes to spatial infinity. That is, the Lorenz condition itself only partially fixes a gauge. The remaining freedom—such as how the potentials behave as one goes to spatial infinity—can be specified in the initial conditions, but no empirical considerstions can dictate what those initial conditions are.

Lorenz gauge is mathematically convenient because in it the dynamical equations for the vector and scalar potentials decouple, which explains why Lorenz would have employed it in 1867, long before any hint of the Theory of Relativity. However, to a more modern eye, something quite different jumps off the page. The d’Alembertian operator is obviously exactly the right form to be invariant under a Lorentz transformation (that’s Hendrik Lorentz). In other words, having decided to take the potentials ontologically seriously, we find that in one gauge the nomology simplifies and shows common structure for the scalar and vector potentials. And if one had the Canonical Presentation of Maxwellian electromagnetism in Lorenz gauge, it might possibly occur to you that the fundamental dynamical equations for the potentials could suggest a change in the spatiotemporal structure. At least as far as the potentials go, all the spatiotemporal structure would need to do is to allow for the definition of the d’Alembertian. By such a route, one could have arrived at Special Relativity—as understood by Minkowski—as a new theory of space-time structure. In short, to a modern sensibility, Maxwell’s theory expressed in Lorenz gauge virtually shouts “I want to live in Minkowski space-time”. This gives some indication of the heuristic power that may accrue to presenting a theory in Canonical form. It becomes clear exactly what is being postulated and how the parts fit together.

We can, of course, play exactly the same game on the new theory as we did on the other theories: instead of the ontologically independent scalar potential and charge density requiring a law in the nomology to keep them correlated, one can just decide to cut the Gordian knot by making the ontological identification of ρ with $\frac{\partial^{2}\;\phi }{\partial t^{2}}-\;\nabla^{2}\phi$. Just as we made the electric charge density into a structural feature of the electric field above, so we can make it a structural feature—indeed, a Lorentz invariant structural feature—of the scalar potential here. That move reduces the whole dynamics of the potentials to one law, as shown below (Table 7).

Of course, in direct parallel to the discussion above, the status of the velocity vector is obscure when using a mass density rather than a particle theory. And the same move to a particle theory is available again.

We are now in a position to make the most important observation so far. By trading off the electric and magnetic fields in the original ontology for the scalar and vector potentials, we have been able to save locality even while handling the Aharonov–Bohm effect: in fact, in the quantum mechanical one-particle theory the wave function of the electron couples to the vector potential in a completely local way. Changing the physical state of the solenoid necessarily changes the vector potential outside the solenoid, so the trick behind the effect seems to be revealed. However, one price we pay for taking the potentials seriously is taking the gauge ontologically seriously as well. What in the classical theory was a merely conventional and unphysical change of gauge becomes the means of changing out one theory in favor of a rival, distinct theory. Our next example illustrates this.

Another of the most popular gauges used in classical Electromagnetism is the Coulomb gauge. The Coulomb gauge condition is $\mathrm{Div}\vec{A}=\;0$. Looking back at the form that Maxwell’s laws take in terms of the potentials, the Coulomb condition again simplifies the nomology. Further, unlike the Lorenz condition, the Coulomb condition is a full gauge-fixing condition: imposing it eliminates all the original freedom in picking a gauge. Hence the resulting theory—once we take the potentials as elements of the physical ontology—is both deterministic and does not require any choice of initial conditions not motivated empirically. We take for granted that the local charge distribution, unlike the value of the scalar or vector potential, is empirically observable.

Here is a naïve attempt at a Canonical Presentation of Classical Electromagnetism formulated in terms of the potentials in Coulomb Gauge. We have eliminated the charge density by ontological definition in terms of the scalar potential (Table 8). To improve readability, we have employed $\vec{j}$ in presenting the nomology, but it can be eliminated in favor of the fundamental Physical Ontology via the Constitutive Principles at will.

A careful examination of the Canonical presentation reveals an anomaly: there are two separate equations that relate the scalar potential ϕ and the charge density ρ. One is the local equation that derives from the desire to eliminate the charge density by definition as first just the Divergence of the electric field and then as the negative of the Laplacian of the scalar potential. This is a local equation in that the scalar potential in any neighborhood of a point determines the charge at that point. The second equation is most naturally read the other way. It defines the scalar potential in a non-local way: the scalar potential is the sum of all the contributions of all the charge densities in the universe with an inverse squared-distance dependency. This definition makes the value of ϕ at any point a function of the contemporaneous charge distribution throughout the universe.

Clearly, one does not want to define the charge distribution in terms of the scalar potential and then turn around and define the scalar potential as a function of the charge distribution. So at best one of the two equations can survive: either the local or the non-local one. Einstein’s choice, of course, would be the local one, but we are going to make the other decision: keep the equation relating the scalar potential to the contemporaneous charge distribution. The next question is the ontological status of this equation. Is it an ontological analysis of charge in terms of scalar potential, an ontological analysis of scalar potential in terms of charge, or an element of the nomology: a law relating the scalar potential to the contemporaneous charge distribution? Again, we will make the choice of treating it as a law, an “instantaneous production-at-a-distance” law. The picture is that the scalar potential is an *effect* of the contemporaneous global charge distribution.

One could obviously make any of these decisions differently, and so end up with a different theory. The whole universe of such theories would be interesting to map, but for reasons that will soon become apparent, this is the articulated theory I want to pursue here.

The Canonical Presentation is seen in Table 9.

Of course, as a theory with matter densities rather than particles, the significance of the velocity that occurs explicitly in the Lorenz Force Law and implicitly in the dynamical equation for the vector potential (via the current) is somewhat obscure. So, as our final adjustment of the theory we will replace the mass density and charge density with point particles that have characteristic masses and charges. That yields Table 10.

There are various ‘t’s to cross and ‘i’s to dot, but let us stop here. So far we have articulated eight separate theories—theories with distinct ontologies and nomologies—based on classical electromagnetic theory. If one uses the phrase “Maxwell’s theory” or “classical electromagnetic theory” in a way that is neutral between all or some of these precisely specified theories then one is using the term “theory” too loosely for metaphysical or ontological purposes. If you want to know what a theory posits about the world then you need to have a theory that is clearly enough articulated to correspond to a single Canonical Presentation.

Note that there is nothing that would have prevented Maxwell from considering and accepting some of these had it occurred to him to “take the potentials seriously”. Note also that of all the theories we have considered yield the same empirical predictions, on any clear notion of “empirical predictions”.

## 6. Adjusting the Spatiotemporal Structure

So far, we have considered ways of changing a theory so that particular equations in the Canonical Presentation get moved around: from the Nomology to the Constitutive Principles of Derivative Ontology, from Mathematical Fictions to Mathematical Representations of Fundamental Ontology, etc. In order to account for the Aharonov–Bohm effect, either we needed something more than the electric and magnetic fields in the ontology or we needed an explicit action-at-a-distance law. Aharonov and Bohm’s original suggestion was that we have to take the potentials more seriously, an idea that can be implemented in many ways (as we have seen). However, the immediate price to pay for changing the status of the potentials is simple: radical indeterminism. One obvious way to get rid of that is to fix gauge. Both the Lorenz and Couloumb conditions fix the gauge enough to eliminate the indeterminism. A weaker reduction in the gauge degrees of freedom, could leave us with radical indeterminism.

However, one thing has remained untouched so far: the spatio-temporal structure. Every one of our theories has been constructed in the same space-time setting: Newtonian Absolute space and Absolute Time. Obviously, this is a component that not only can but must be adjusted to account for Relativistic effects in a plausible way. Our final topic is reflection on this situation.

Just as the physical ontology and the nomology are mutually constraining—only mathematical representations of items in the physical ontology should appear in the nomology and every such representation should appear somewhere in the nomology—so too are the contents of the nomology and the spatio-temporal structure. You need enough spatio-temporal structure to express the laws, and don’t want more structure than is required for that purpose. Having the full Absolute Space and Time gives one a lot of structure to work with, and indeed more structure than one needs. In particular, it allows one to define the absolute velocity of a particle, which is a notion that has long been regarded as suspect. Playing the same game with Newtonian mechanics reveals that some of the structure of Absolute Space and Time is otiose for that theory, and one can set a similar theory in Galilean space-time. What considerations apply to the question of spatio-temporal structure here?

If we set r(x, y, z, t) or all the q_i_ to zero, all that is left of Maxwell’s theory is the homogeneous Maxwell equations. In the theory that makes the potentials fundamental, choosing the Lorenz gauge condition converts the nomology to ${□}^{2}\phi =\;0$ and ${□}^{2}\vec{A}=0$. As noted above, if this where the whole story ended then the theory would be suggesting that it lives naturally in a Minkowski space. The d’Almerbertian is easily and naturally definable in Minkowski space-time, where it is Lorentz invariant. Indeed, the Lorentz invariance of the nomology in Lorenz gauge is one of the reasons so many people refer to Lorenz gauge as Lorentz gauge. Even more importantly, the manifest Lorentz invariance of the theory cast in Lorenz gauge provides an easy argument to the conclusion that Maxwellian electro-magnetic is a Lorentz invariant theory. One should then switch the spatio-temporal structure to Minkowski, as it is simpler.

There are a few flies in the ointment. One is that the homogeneous equations are not the whole story. Charged matter does have to be introduced into the theory somehow, so we need an updated version of the Lorentz force law and a dynamics to go with it. The current also needs to be reintroduced and the dynamical equation for the vector potential made sensitive to the matter. Nonetheless, the very existence of the Lorenz gauge has convinced physicists to regard Maxwell’s theory as implicitly Relativistic.

Another fly, concerns the fact that the Lorenz condition makes the exact value of the vector potential empirically inaccessible. This can be settled in an island universe by imposing a demand on how the potential behaves as it goes to infinity, but what is the physical motivation for such a constraint?. So long as the potentials were regarded as mere mathematical fictions this made no difference, and the potentials in Lorenz gauge could be used to prove the Lorentz invariance of the field theory. However, if the potentials are not mere fictions, then we would prefer a deterministic theory with empirically justified attribution of values to the potentials.

These reflections suggest a different conclusion: instead of Lorenz gauge, consider a theory in which the potentials are subject to the Coulomb condition. Now the gauge is completely fixed given the charge distribution, yielding both determinism and empirical justification of the initial conditions. What sort of spatiotemporal structure would be needed to express the nomology of this theory?

The presence of the d’Alembertian in the equation for the vector field once again suggests a Minkowski space-time structure. But Coulomb’s Law for the scalar potential points in a very different direction. Since the scalar potential, in Coulomb gauge, is a function of the contemporaneous charge distribution throughout all space, one requires a structure akin to Absolute Simultaneity in order to define the theory. It is natural, from this point of view, to add such a structure—a preferred foliation—to the Minkowski space-time.

By this line of argument, merely noticing the Aharonov–Bohm effect, without having a clue about where it originates, could motivate replacing Absolute Space and Absolute Time with a Lorentzian space-time plus a preferred foliation. This suggestion has arisen without any consideration of experimental violation of Bell’s inequality. To review the argument: the Aharonov–Bohm phenomenon suggests that the electromagnetic situation outside of the solenoid must change when the flux inside changes. But according to the field theory, the fields outside do not change at all. The vector potential, however, does. So we reify the vector and scalar potentials. Now when the flux changes inside the solenoid the vector potential outside *must* change since the Curl of it has changed inside (via Stokes’ Law). So by reifying the potentials we provide the resources needed (ultimately by quantum mechanics) to account for the effect using only local interaction of the vector potential and the electron quantum state. However, in Coulomb gauge, one posits at the very same time a non-local law relating the charge distribution on a leaf of the preferred foliation to the value of the scalar field there. This combination of Relativistic locality with foliation-dependent non-locality is at least strongly suggested by taking the Coulomb condition seriously. It is worthy of both careful consideration and astonishment that the corresponding space-time structure is precisely what one needs to adapt Bohmian mechanics to a Relativistic setting.

It is worthwhile to reflect on this astonishing fact. A little counterfactual history illustrates the point. Suppose that, before the development of quantum theory, an experimentalist just stumbled on the two-slit interference phenomenon. Then again, quite by accident, the experimentalist stuck a solenoid in the experimental design and discovered the Aharonov–Bohm effect. Armed only with classical Maxwellian electro-magnetism in a classical space-time and the familiar Maxwellian calculational techniques, what conclusions might such a physicist entertain?

Without any prompting, the experimentalist would notice that there are shifts in the interference bands even though the electric and magnetic fields outside the solenoid are unchanged. Wary of action-at-a-distance, the experimentalist would look for some physical magnitude that does change outside the solenoid. It is not far to seek in the mathematics: when the magnetic flux in the solenoid changes, the vector potential outside the solenoid must of necessity change too: Gauss’s Law demands it. So the experimentalist would first be enticed by Aharonov and Bohm’s conclusion: rather than merely being mathematical conveniences for solving problems, the mathematical scalar and vector potentials directly represent something physically real!

However, now the specter of gauge freedom raises its head. Our physicist has become accustomed to choosing whatever gauge equivalent vector and scalar potentials happen to be most convenient for the problem at hand, and has done so with a clear conscience because the potentials were regarded as just mathematical fictions, mere conveniences. “Making them real”, whatever that precisely may come to mean, makes the conscience uneasy. If a changed vector potential in a region with an unchanged electric and magnetic field can make an observable difference, then the choice of a gauge cannot just be written off as unproblematic.

We now imagine a chain of possible reactions (not the only possible ones by any means!). First, it occurs to the physicists to cut out the former gauge degrees of freedom by gauge fixing. However, Alice chooses Lorenz gauge and Bob Coulumb gauge. Each puts the appropriate fundamental equation into the nomology, and asks: “What sort of a space-time structure do I really need to make sense of these equations?”. Alice has chosen Lorenz gauge, and is now paying much more attention to it. She sees that the equations for A and phi decouple, and furthermore that the decoupled equations both have the form of a d’Alembertian acting on the potential. Noting that the d’Alembertian looks just like a Laplacian with an extra term that has flipped parity, we have already come perilously close to considering the Minkowski metric and what it could describe. So we have the familiar straight-line route from Maxwellian E & M to Special Relativity.

Meanwhile, Bob always liked to work in Coulomb gauge, so he imposes it as the condition on A and phi. Now there are two interlocked equations, one using the d’Alembertian again and the other a simple instantaneous action-at-a-distance formula for the electric potential. Having heard about Alice’s work, the d’Alembertian suggests a Minkowski space-time. However, the phi dynamics require, laid over this space-time, a physical foliation. Thus we arrive at a picture of the space-time structure containing both a Relativistic metric and a privileged set of level surfaces.

The Aharonov–Bohm effect does not violate any Bell’s Inequality, and can be explained in a completely local way as Alice’s theory shows. Nonetheless, the extensive use of Coulomb gauge makes a natural opening—once you decide to reify the vector and scalar potentials—for a space-time structure with both a Lorentzian metric and a preferred foliation. It is exactly that space-time structure that makes it easy to explain Relativistic effects in Bohmian Mechanics, as well as to implement the non-locality that we know any empirically successful theory requires.

Our examination of various proposals for how to account for the Aharonov–Bohm effect by altering the fundamental ontology and/or nomology of the Maxwellian electrodynamics has been for illustrative rather than substantial purposes. As we have seen, even before trying to take quantum theory explicitly into account and operating in a purely classical setting, there are many, many options. We have only just touched on the changes in spatio-temporal structure that Relativity introduces. Further, the effect itself has more complex and subtle forms (see, for example, [9,10]). There is a tremendous amount of detailed work to be done in order to really come to grips with the ultimate ontology of what Maxwell thought of as the electric and magnetic fields. This essay has been concerned with what the general nature of that work is, and how it can be pellucidly displayed, not with what the ultimate outcome should be.

## 7. Conclusions

Having seen how many different ways the basic structure of classical electromagnetism can be used when constructing alternative, precisely defined theories (as articulated by a Canonical Presentation) it may come as no surprise that we have barely scratched the surface. Electromagnetic theory can, for example, be reformulated in the mathematical language of fiber bundles. In that setting, the fields are derivative, corresponding to the curvature of the connection on the bundle [2]. That mathematical formalism is suggestive of yet another bevy of precise physical theories that the Canonical Presentation could help keep straight.

If physicists were to adopt this method—or any other standardized method—to convey physical theories clearly and unambiguously, many conceptual problems could be avoided. First and foremost is the unfortunate tendency to portray different theories as nothing but different “interpretations” of one and the same theory. Further, the discipline that a standardized format of this sort imposes can make it easier to notice alterative theories that have not yet been considered.

Talk of physical ontology vs. nomology; of derivative ontology vs. mathematical fiction; of spatio-temporal structure; and of fundamentality may strike one as philosophical rather than physical. But these sorts of distinctions lie at the heart of physics, even if they are not often acknowledged. Aharonov and Bohm recognized this perfectly well, so it seems apt to give the last word to them:

In classical mechanics, we recall that potentials cannot have such significance because the equation of motion involves only the field quantities themselves. For this reason, the potentials have been regarded as purely mathematical auxiliaries, while only the field quantities were thought to have a direct physical meaning.

In quantum mechanics, the essential difference is that the equations of motion for a particle are replaced by the Schrödinger equation for a wave. This Schrödinger equation is obtained from a canonical formula, which cannot be expressed in terms of the fields alone, but which also requires the potentials. Indeed, the potentials play a role, in Schrödinger’s equation, which is analogous to that of the index of refraction in optics. The Lorentz force [*e***E** + (*e*/*c*)**v** × **H**] does not appear anywhere in the fundamental theory, but appears only as an approximation appearing in the classical limit. It would therefore seem natural at this point to propose that, in quantum mechanics, the fundamental physical entities are the potentials, while the fields are derived from by differentiations.

([1], p. 490)

## Figures and Tables

**Table 1:** Standard E-M Theory.

Theory	Physical Ontology; Spatiotemporal Structure	Mathematical Representation of Physical Ontology	Purely Mathematical Facts	Nomology	Derivative Ontology; Mathematical Fictions
Classical E & M, Mass Density Version	Electric Field Magnetic Field Charge Density Mass Density Lorentz Force; Time 3-D Euclidean Absolute Space	$\vec{E}$(x, y, z, t) $\vec{B}$(x, y, z, t) ρ(x, y, z, t) μ(x, y, z, t) $\vec{F_{L}}$(x, y, z, t) t ∈ ℝ (x, y, z) ∈ ℝ ^3^	If Curl $\vec{C}$ = 0 on a simply connected space, then $\vec{C}=\mathrm{Grad}\left(\xi \right)$ for some ξ. If Div $\vec{B}$ = 0 on a simply connected space, then $\vec{B}=\mathrm{Curl}\left(\vec{A}\right)$ for some $\vec{A}$ Gauge transformations $\vec{A\prime }=\;\vec{A}+\;\mathrm{grad}\xi$ $\phi^{\prime }=\;\phi -\frac{\partial \xi }{\partial t}$	$\mathrm{Div}\left(\vec{E}\right)=\;\rho$ $\mathrm{Div}\left(\vec{B}\right)=\;0$ $\mathrm{Curl}\left(\vec{E}\right)+\;\frac{\partial \vec{B}}{\partial t}=0$ $\mathrm{Curl}\left(\vec{B}\right)-\;\frac{\partial \vec{E}}{\partial t}=\rho \vec{v}$ $\vec{F_{L}}=\;\rho \left(\vec{E}+\left(\vec{v}\;\times \;\vec{B}\right)\right)$ $\vec{F_{\mathrm{net}}}=\;\mu \frac{d\vec{v}}{dt}$	Derivative Ontology: $\vec{J}=\;\rho \vec{v}$ $\vec{F_{\mathrm{net}}}$ = vector sum of all forces on a body at a point Mathematical Fictions: Let $\vec{B}=\mathrm{Curl}\left(\vec{A}\right)$ Then $\mathrm{Curl}\left(\vec{E}+\;\frac{\partial \vec{A}}{\partial t}\right)=0$ so $\vec{E}+\;\frac{\partial \vec{A}}{\partial t}=-\mathrm{Grad}\left(\phi \right)$ or $\vec{E}=-\mathrm{Grad}\left(\phi \right)$ $–\;\frac{\partial \vec{A}}{\partial t}$

**Table 2:** E-M with a Particle Ontology.

Theory	Physical Ontology; Spatiotemporal Structure	Mathematical Representation of Physical Ontology	Purely Mathematical Facts	Nomology	Derivative Ontology; Mathematical Fictions
Classical E & M, Particle Version	Electric Field Magnetic Field Point Particles Particle Charge Particle Mass Lorentz Force; Time 3-D Euclidean Absolute Space	$\vec{E}$(x,y,z,t) $\vec{B}$(x,y,z,t) $\vec{x_{i}}$(t) = (x_i_,y_i_,z_i_) q_i_∈ R m_i_ ∈ R > 0 $\vec{F_{L}}$(x,y,z,t) t ∈ ℝ (x,y,z) ∈ ℝ ^3^	If Curl $\vec{C}$ = 0 on a simply connected space, then $\vec{C}=\mathrm{Grad}\left(\xi \right)$ for some ξ. If Div $\vec{B}$ = 0 on a simply connected space, then $\vec{B}=\mathrm{Curl}\left(\vec{A}\right)$ for some $\vec{A}$ Gauge transformations $\vec{A\prime }=\;\vec{A}+\;\mathrm{grad}\xi$ $\phi^{\prime }=\;\phi -\frac{\partial \xi }{\partial t}$	$\mathrm{Div}\left(\vec{E}\right)=\;q_{i}$ $\mathrm{Div}\left(\vec{B}\right)=\;0$ $\mathrm{Curl}\left(\vec{E}\right)+\;\frac{\partial \vec{B}}{\partial t}=0$ $\mathrm{Curl}\left(\vec{B}\right)-\;\frac{\partial \vec{E}}{\partial t}=q_{i}\vec{v_{i}}$ $\vec{F_{Li}}=\;q_{i}\left(\vec{E}+\left(\vec{v_{i}}\;\times \;\vec{B}\right)\right)$ $\vec{F_{\mathrm{net}i}}=\;m_{i}\frac{d^{2}\vec{x_{i}}\;\left(t\right)}{dt^{2}}$	Derivative Ontology: $\vec{v_{i}}=\frac{d\vec{x_{i}}\;\left(t\right)}{dt}$ $\vec{J_{i}}=\;q_{i}\vec{v_{i}}$ $\vec{F_{\mathrm{net}}}$ = vector sum of all forces on a particle Mathematical Fictions: Let $\vec{B}=\mathrm{Curl}\left(\vec{A}\right)$ Then $\mathrm{Curl}\left(\vec{E}+\;\frac{\partial \vec{A}}{\partial t}\right)=0$ so $\vec{E}+\;\frac{\partial \vec{A}}{\partial t}=-\mathrm{Grad}\left(\phi \right)$ or $\vec{E}=-\mathrm{Grad}\left(\phi \right)$ $-\;\frac{\partial \vec{A}}{\partial t}$

**Table 3:** E-M with Derived Charges.

Theory	Physical Ontology; Spatiotemporal Structure	Mathematical Representation of Physical Ontology	Purely Mathematical Facts	Nomology	Derivative Ontology; Mathematical Fictions
Classical E & M, Particle Version With Derived Charges	Electric Field Magnetic Field Point Particles Particle Mass Lorentz Force; Time 3-D Euclidean Absolute Space	$\vec{E}$(x, y, z, t) $\vec{B}$(x, y, z, t) $\vec{x_{i}}$(t) = (x_i_, y_i_, z_i_) m_i_∈ R > 0 $\vec{F_{L}}$(x, y, z, t); t ∈ ℝ (x, y, z) ∈ ℝ ^3^	If Curl $\vec{C}$ = 0 on a simply connected space, then $\vec{C}=\mathrm{Grad}\left(\xi \right)$ for some ξ. If Div $\vec{B}$ = 0 on a simply connected space, then $\vec{B}=\mathrm{Curl}\left(\vec{A}\right)$ for some $\vec{A}$ Gauge transformations $\vec{A\prime }=\;\vec{A}+\;\mathrm{grad}\xi$ $\phi^{\prime }=\;\phi -\frac{\partial \xi }{\partial t}$	$\mathrm{Curl}\left(\vec{E}\right)+\;\frac{\partial \vec{B}}{\partial t}=0$ $\mathrm{Curl}\left(\vec{B}\right)-\;\frac{\partial \vec{E}}{\partial t}=q_{i}\vec{v_{i}}$ $\vec{F_{Li}}=\;q_{i}\left(\vec{E}+\left(\vec{v_{i}}\;\times \;\vec{B}\right)\right)$ $\vec{F_{\mathrm{net}i}}=\;m_{i}\frac{d^{2}\vec{x_{i}}\;\left(t\right)}{dt^{2}}$	Derivative Ontology: $\mathrm{Div}\left(\vec{E}\right)=\;q_{i}$ $\mathrm{Div}\left(\vec{B}\right)=\;q_{m}$ $\vec{v_{i}}=\frac{d\vec{x_{i}}\;\left(t\right)}{dt}$ $\vec{J_{i}}=\;q_{i}\vec{v_{i}}$ $\vec{F_{\mathrm{net}}}$ = vector sum of all forces on a particle Mathematical Fictions: Let $\vec{B}=\mathrm{Curl}\left(\vec{A}\right)$ Then $\mathrm{Curl}\left(\vec{E}+\;\frac{\partial \vec{A}}{\partial t}\right)=0$ so $\vec{E}+\;\frac{\partial \vec{A}}{\partial t}=-\mathrm{Grad}\left(\phi \right)$ or $\vec{E}=-\mathrm{Grad}\left(\phi \right)$ $-\;\frac{\partial \vec{A}}{\partial t}$

**Table 4:** Particle Ontology with derived charge.

Theory	Physical Ontology; Spatiotemporal Structure	Mathematical Representation of Physical Ontology	Purely Mathematical Facts	Nomology	Derivative Ontology; Mathematical Fictions
Classical E & M, Particle Version With Derived Charges	Electric Field Magnetic Field Point Particles Particle Mass Lorentz Force; Time 3-D Euclidean Absolute Space	$\vec{E}$(x, y, z, t) $\vec{B}$(x, y, z, t) s.t. $\mathrm{Div}\left(\vec{B}\right)=\;0$ $\vec{x_{i}}$(t) = (x_i_, y_i_, z_i_) m_i_∈ R > 0 $\vec{F_{L}}$(x, y, z, t) t ∈ ℝ (x, y, z) ∈ ℝ ^3^	If Curl $\vec{C}$ = 0 on a simply connected space, then $\vec{C}=\mathrm{Grad}\left(\xi \right)$ for some ξ. If Div $\vec{B}$ = 0 on a simply connected space, then $\vec{B}=\mathrm{Curl}\left(\vec{A}\right)$ for some $\vec{A}$ Gauge transformations $\vec{A\prime }=\;\vec{A}+\;\mathrm{grad}\xi$ $\phi^{\prime }=\;\phi -\frac{\partial \xi }{\partial t}$	$\mathrm{Curl}\left(\vec{E}\right)+\;\frac{\partial \vec{B}}{\partial t}=0$ $\mathrm{Curl}\left(\vec{B}\right)-\;\frac{\partial \vec{E}}{\partial t}=q_{i}\vec{v_{i}}$ $\vec{F_{Li}}=\;q_{i}\left(\vec{E}+\left(\vec{v_{i}}\;\times \;\vec{B}\right)\right)$ $\vec{F_{\mathrm{net}i}}=\;m_{i}\frac{d^{2}\vec{x_{i}}\;\left(t\right)}{dt^{2}}$	Derivative Ontology: $\mathrm{Div}\left(\vec{E}\right)=\;q_{i}$ $\vec{v_{i}}=\frac{d\vec{x_{i}}\;\left(t\right)}{dt}$ $\vec{J_{i}}=\;q_{i}\vec{v_{i}}$ $\vec{F_{\mathrm{net}}}$ = vector sum of all forces on a particle Mathematical Fictions: Let $\vec{B}=\mathrm{Curl}\left(\vec{A}\right)$ Then $\mathrm{Curl}\left(\vec{E}+\;\frac{\partial \vec{A}}{\partial t}\right)=0$ so $\vec{E}+\;\frac{\partial \vec{A}}{\partial t}=-\mathrm{Grad}\left(\phi \right)$ or $\vec{E}=-\mathrm{Grad}\left(\phi \right)$ $-\;\frac{\partial \vec{A}}{\partial t}$

**Table 5:** Naïve Theory with Ontology of Potentials.

Theory	Physical Ontology; Spatiotemporal Structure	Mathematical Representation of Physical Ontology	Purely Mathematical Facts	Nomology	Constitutive Principles of Derivative Ontology
Vector and scalar potentials, Mass density, Newtonian Space and Time	Vector Potential Scalar Potential Charge density Mass density Lorentz Force; Time 3-D Euclidean Absolute Space	$\vec{A}$(x, y, z, t) ϕ(x, y, z, t) ρ(x, y, z, t) μ(x, y, z, t) $\vec{F_{L}}$(x, y, z, t) t ∈ ℝ (x, y, z) ∈ ℝ ^3^	The nomology does not fix the history of $\vec{A},\;\mathrm{or}\;\phi$, given complete initial values. Radical indeterminism $\vec{A\prime }=\;\vec{A}+\;\mathrm{Grad}\xi$ $\phi^{\prime }=\;\phi -\frac{\partial \xi }{\partial t}$	$\nabla^{2}\phi -\;\frac{\partial \;\mathrm{Div}\;\vec{A}}{\partial t}=-\;\rho$ $\nabla^{2}\vec{A}-\frac{\partial^{2}\vec{A}}{\partial t^{2}}-\;\mathrm{Grad}\left(\mathrm{Div}\vec{A}+\;\frac{\partial \phi }{\partial t}\right)=\rho \vec{v}$ $\vec{F_{\mathrm{net}}}=\;\mu \frac{d\vec{v}}{dt}$ $\vec{F_{L}}=\rho \left(-\mathrm{Grad}\phi -\frac{d\vec{A}}{dt}+\mathrm{Grad}\left(\vec{v}\;\cdot \;\vec{A}\right)\right)$	$\vec{j}=\;\rho \vec{v}$ $\vec{B}=\mathrm{Curl}\;\vec{A}$ $\vec{E}=-\mathrm{Grad}\left(\phi \right)$ $-\;\frac{\partial \vec{A}}{\partial t}$

**Table 6:** Lorenz-Gauge Theory with Charge Density.

Theory	Physical Ontology; Spatiotemporal Structure	Mathematical Representation of Physical Ontology	Purely Mathematical Facts	Nomology	Constitutive Principles of Derivative Ontology
Vector and scalar potentials in Lorenz Gauge, Mass density, Charge density Newtonian Space and Time	Vector Potential Scalar Potential Charge density Mass density Lorentz Force; Time 3-D Euclidean Absolute Space	$\vec{A}$(x, y, z, t) ϕ(x, y, z, t) ρ(x, y, z, t) μ(x, y, z, t) $\vec{F_{L}}$(x, y, z, t) t ∈ ℝ (x, y, z) ∈ ℝ ^3^	Definition of Lorenz Gauge: $\mathrm{Div}\vec{A}=-\;\frac{\partial \phi }{\partial t}$ ϕ is fixed by initial boundary conditions, e.g., requiring it to zero sufficiently fast at ∞.	$\nabla^{2}\phi -\;\frac{\partial^{2}\;\phi }{\partial t^{2}}=-\;\rho$ $\nabla^{2}\vec{A}-\;\frac{\partial^{2}\;\vec{A}}{\partial t^{2}}=-\rho \vec{v}$ or, using the d’Alembertian: ${□}^{2}\;\phi =-\;\rho$ ${□}^{2}\vec{A}=-\rho \vec{v}$ $\mu \frac{d\vec{v}}{dt}=\rho \left(-\mathrm{Grad}\phi -\frac{d\vec{A}}{dt}+\mathrm{Grad}\left(\vec{v}\;\cdot \;\vec{A}\right)\right)$ (if $\vec{F_{L}}=\vec{F_{\mathrm{net}}}$)	$\vec{j}=\;\rho \vec{v}$ $\vec{B}=\mathrm{Curl}\;\vec{A}$ $\vec{E}=-\mathrm{Grad}\left(\phi \right)$ $-\;\frac{\partial \vec{A}}{\partial t}$

**Table 7:** Lorenz Gauge, Derived Charge Density.

Theory	Physical Ontology; Spatiotemporal Structure	Mathematical Representation of Physical Ontology	Purely Mathematical Facts	Nomology	Constitutive Principles of Derivative Ontology
Vector and scalar potentials in Lorenz Gauge, Mass density, Derived Charge Density Newtonian Space and Time	Vector Potential Scalar Potential Mass density Lorentz Force; Time 3-D Euclidean Absolute Space	$\vec{A}$(x, y, z, t) ϕ(x, y, z, t) μ(x, y, z, t) $\vec{F_{L}}$(x, y, z, t) t ∈ ℝ (x, y, z) ∈ ℝ ^3^	Definition: Lorenz Gauge $\mathrm{Div}\vec{A}=-\;\frac{\partial \phi }{\partial t}$ ϕ is also gauge-fixed if it has to go to zero sufficiently fast at ∞.	$\nabla^{2}\vec{A}-\;\frac{\partial^{2}\;\vec{A}}{\partial t^{2}}=-\rho \vec{v}$ or, using the d’Alembertian: ${□}^{2}\vec{A}=-\rho \vec{v}$ $\mu \frac{d\vec{v}}{dt}={□}^{2}\phi \left(-\mathrm{Grad}\phi -\frac{d\vec{A}}{dt}+\mathrm{Grad}\left(\vec{v}\;\cdot \;\vec{A}\right)\right)$ (if $\vec{F_{L}}=$$\vec{F_{\mathrm{net}}}$)	$\vec{j}=\;\rho \vec{v}$ $\vec{B}=\mathrm{Curl}\;\vec{A}$ $\vec{E}=-\mathrm{Grad}\left(\phi \right)$$-\;\frac{\partial \vec{A}}{\partial t}$ $\nabla^{2}\phi -\;\frac{\partial^{2}\;\phi }{\partial t^{2}}=-\;\rho$ or $\rho =-{□}^{2}\phi$

**Table 8:** A Naïve Attempt at Coulomb-fixed Potentials.

Theory	Physical Ontology; Spatiotemporal Structure	Mathematical Representation of Physical Ontology	Purely Mathematical Facts	Nomology	Constitutive Principles of Derivative Ontology
Vector and scalar potentials in Coulomb Gauge, Mass density, Charge Density Newtonian Space and Time	Vector Potential Scalar Potential Mass Density Charge Density Lorentz Force; Time 3-D Euclidean Absolute Space	$\vec{A}$(x, y, z, t) ϕ(x, y, z, t) μ(x, y, z, t) ρ(x, y, z, t) $\vec{F_{L}}$(x, y, z, t) t ∈ ℝ (x, y, z) ∈ ℝ ^3^	Definition of Coulomb Gauge $\mathrm{Div}\vec{A}=0$	$\;\frac{\partial^{2}\;\vec{A}}{\partial t^{2}}-\nabla^{2}\vec{A}=\vec{j}-\;\mathrm{Grad}\left(\;\frac{\partial \phi }{\partial t}\right)$ or, using the d’Alembertian: $-{□}^{2}\vec{A}=\vec{j}-\;\mathrm{Grad}\left(\;\frac{\partial \phi }{\partial t}\right)$ $\mu \frac{d\vec{v}}{dt}=-\nabla^{2}\phi \;\left(\mathrm{Grad}\left(\phi -\left(\vec{v}\;\cdot \;\vec{A}\right)\right)+\frac{d\vec{A}}{dt}\right)$ (if $\vec{F_{L}}=$$\vec{F_{\mathrm{net}}}$) $\phi \left(\vec{x},t\right)=\int^{\text{​}}\frac{\rho \left(\vec{x\prime },t\right)}{\left|\vec{x}-\vec{x\prime }\right|}d^{3}\vec{x\prime }$	$\vec{j}=\;\rho \vec{v}$ $\vec{B}=\mathrm{Curl}\;\vec{A}$ $\vec{E}=-\mathrm{Grad}\left(\phi \right)$ $-\;\frac{\partial \vec{A}}{\partial t}$ $-\nabla^{2}\phi =\;\rho$

**Table 9:** Coulomb Condition, Derived Charge Density.

Theory	Physical Ontology; Spatiotemporal Structure	Math Representation of Physical Ontology	Purely Mathematical Facts	Nomology	Constitutive Principles of Derivative Ontology
Vector and scalar potentials in Coulomb Gauge, Mass density, Derived Charge Density Newtonian Space and Time	Vector Potential Scalar Potential Mass density Lorentz Force; Time 3-D Euclidean Absolute Space	$\vec{A}$(x, y, z, t) ϕ(x, y, z, t) μ(x, y, z, t) $\vec{F_{L}}$(x, y, z, t) t ∈ ℝ (x, y, z) ∈ ℝ ^3^	Definition of Coulomb Gauge $\mathrm{Div}\vec{A}=0$ $\int^{\text{​}}\frac{\rho \left(\vec{x\prime },t\right)}{|\vec{x}-\vec{x\prime }|\prime }d^{3}\vec{x\prime }$	$\;\frac{\partial^{2}\;\vec{A}}{\partial t^{2}}-\nabla^{2}\vec{A}=\vec{j}-\;\mathrm{Grad}\left(\;\frac{\partial \phi }{\partial t}\right)$ or, using the d’Alembertian: $-{□}^{2}\vec{A}=\vec{j}-\;\mathrm{Grad}\left(\;\frac{\partial \phi }{\partial t}\right)$ $\mu \frac{d\vec{v}}{dt}=-\nabla^{2}\phi \;\left(\mathrm{Grad}\left(\phi -\left(\vec{v}\;\cdot \;\vec{A}\right)\right)+\frac{d\vec{A}}{dt}\right)$ (if $\vec{F_{L}}=$ $\phi \left(\vec{x},t\right)=$ $\vec{F_{\mathrm{net}}}$)	$\vec{j}=\;\rho \vec{v}$ $\vec{B}=\mathrm{Curl}\;\vec{A}$ $\vec{E}=-\mathrm{Grad}\left(\phi \right)$ $-\;\frac{\partial \vec{A}}{\partial t}$ $-\nabla^{2}\phi =\;\rho$

**Table 10:** Potentials with Coulomb Condition and Particles.

Theory	Physical Ontology; Spatiotemporal Structure	Mathematical Representation of Physical Ontology	Purely Mathematical Facts	Nomology	Constitutive Principles of Derivative Ontology
Vector and scalar potentials in Coulomb Gauge, Particles with Mass and Charge, Newtonian Space and Time	Vector Potential Scalar Potential Point Particles Particle Charge Particle Mass Lorentz Force Time 3-D Euclidean Absolute Space	$\vec{A}$(x, y, z, t) ϕ(x, y, z, t) $\vec{x_{i}}$(t) = (x_i_, y_i_, z_i_) q_i_∈ R m_i_ ∈ R > 0 $\vec{F_{L}}$(x, y, z, t) t ∈ ℝ (x, y, z) ∈ ℝ ^3^	Definition: Coulomb Gauge $\mathrm{Div}\vec{A}=0$	$\;\frac{\partial^{2}\;\vec{A}}{\partial t^{2}}-\nabla^{2}\vec{A}=\vec{j}-\;\mathrm{Grad}\left(\;\frac{\partial \phi }{\partial t}\right)$ or, using the d’Alembertian: $-{□}^{2}\vec{A}=\vec{J}-\;\mathrm{Grad}\left(\;\frac{\partial \phi }{\partial t}\right)$ $m_{i}\frac{d\vec{v_{i}}}{dt}=q_{i}\left(\mathrm{Grad}\left(\phi -\left(\vec{v_{i}}\;\cdot \;\vec{A}\right)\right)+\frac{d\vec{A}}{dt}\right)$ (if $\vec{F_{L}}=$$\vec{F_{\mathrm{net}}}$) $\phi \left(\vec{x},t\right)=\overset{n=N}{\underset{n=1}{\sum }}\;\frac{q_{i}}{|\vec{x}-\vec{x\prime }|\prime }$ omitting points on particle worldlines	$\vec{j}=\;\rho \vec{v}$ $\vec{B}=\mathrm{Curl}\;\vec{A}$ $\vec{E}=-\mathrm{Grad}\left(\phi \right)$ $-\;\frac{\partial \vec{A}}{\partial t}$ $\vec{v}=\;\vec{v_{i}}=\frac{d\vec{x_{i}}\;\left(t\right)}{dt}$ $\vec{J_{i}}=\;q_{i}\vec{v_{i}}$
